# Immunometabolic capacities of nutritional fatty acids in regulation of inflammatory bone cell interaction and systemic impact of periodontal infection

**DOI:** 10.3389/fimmu.2023.1213026

**Published:** 2023-09-06

**Authors:** Annika Döding, Svenja Zimmermann, Ahmed Maghames, Michael Reimann, Judit Symmank, Maria Thürmer, Markus H. Gräler, Michael Wolf, Collin Jacobs, Andreas Koeberle, Bernd Sigusch, Ulrike Schulze-Späte

**Affiliations:** ^1^ Section of Geriodontics, Department of Conservative Dentistry and Periodontics, University Hospital Jena, Jena, Germany; ^2^ Department of Orthodontics, University Hospital Jena, Jena, Germany; ^3^ Chair of Pharmaceutical and Medicinal Chemistry, Institute of Pharmacy, Friedrich-Schiller-University Jena, Jena, Germany; ^4^ Department of Anesthesiology and Intensive Care Medicine, Center for Molecular Biomedicine (CMB) and Center for Sepsis Control and Care (CSCC), Jena University Hospital, Jena, Germany; ^5^ Department of Orthodontics, University Hospital RWTH Aachen, Aachen, Germany; ^6^ Michael Popp Institute and Center for Molecular Biosciences Innsbruck (CMBI), University of Innsbruck, Innsbruck, Austria; ^7^ Department of Conservative Dentistry and Periodontics, University Hospital Jena, Jena, Germany

**Keywords:** bone remodeling, regeneration, lipids, inflammation, nutrition, bone loss, periodontal disease

## Abstract

**Introduction:**

Novel preventive strategies in periodontal disease target the bacterial-induced inflammatory host response to reduce associated tissue destruction. Strategies focus on the modulation of tissue-destroying inflammatory host response, particularly the reduction of inflammation and promotion of resolution. Thereby, nutrition is a potent immunometabolic non-pharmacological intervention. Human studies have demonstrated the benefit of olive oil-containing Mediterranean-style diets (MDs), the main component of which being mono-unsaturated fatty acid (FA) oleic acid (OA (C18:1)). Hence, nutritional OA strengthened the microarchitecture of alveolar trabecular bone and increased circulating pro-resolving lipid mediators following bacterial inoculation with periodontal pathogen *Porphyromonas gingivalis*, contrary to saturated FA palmitic acid (PA (C16:0)), which is abundant in Western-style diets. Additionally, the generalized distribution of inflammatory pathway mediators can occur in response to bacterial infection and compromise systemic tissue metabolism and bone homeostasis distant from the side of infection. Whether specific FA-enriched nutrition and periodontal inoculation are factors in systemic pathology that can be immune-modulatory targeted through dietary substitution is unknown and of clinical relevance.

**Methods:**

Normal-weight C57BL/6-mice received OA-or PA-enriched diets (PA-ED, OA-ED, PA/OA-ED) or a normal-standard diet (n=12/group) for 16 weeks and were orally infected with *P. gingivalis*/placebo to induce periodontal disease. Using histomorphometry and LC-MS/MS, systemic bone morphology, incorporated immunometabolic FA-species, serological markers of bone metabolism, and stress response were determined in addition to bone cell inflammation and interaction *in vitro*.

**Results:**

In contrast to OA-ED, PA-ED reduced systemic bone microarchitecture paralleled by increased lipotoxic PA-containing metabolite accumulation in bone. Substitution with OA reversed the bone-destructive impact of PA, which was accompanied by reduced diacylglycerols (DAG) and saturated ceramide levels. Further, PA-associated reduction in mineralization activity and concomitant pro-inflammatory activation of primary osteoblasts were diminished in cultures where PA was replaced with OA, which impacted cellular interaction with osteoclasts. Additionally, PA-ED increased osteoclast numbers in femurs in response to oral *P. gingivalis* infection, whereas OA-ED reduced osteoclast occurrence, which was paralleled by serologically increased levels of the stress-reducing lipokine PI(18:1/18:1).

**Conclusion:**

OA substitution reverses the bone-destructive and pro-inflammatory effects of PA and eliminates incorporated lipotoxic PA metabolites. This supports Mediterranean-style OA-based diets as a preventive intervention to target the accumulation of PA-associated lipotoxic metabolites and thereby supports systemic bone tissue resilience after oral bacterial *P. gingivalis* infection.

## Introduction

Nutrition can act as a modifiable immunometabolic key factor in the regulation of non-communicable inflammatory diseases that are determined by metabolic and inflammatory dysregulation (1, 2). Thereby, nutritional concepts target host response and are classified based on their inflammatory potential, which could foster the resolution of inflammation and influence tissue homeostasis (2).

Oral health is tightly connected with other non-communicable diseases (NCD) (3). Specifically, periodontal disease (PD) is becoming a global health problem with an economic burden due to its increasing prevalence and demographic development over the past years (4). PD is an oral inflammatory disease in which a bacterial insult initiates immune-regulated canonical pathways that ultimately result in the destruction of hard and soft tissue (5). A dysbiosis-driven hyper-inflammatory host response, tissue-specific immunometabolic stress responses, and failure in the resolution of inflammation are important determinants of periodontal tissue loss (6). Additionally, the generalized spread of causative bacteria, bacterial products, and mediators of non-resolving inflammatory pathways could compromise systemic tissue homeostasis. Hence, locally produced pro-inflammatory cytokines such as tumor necrosis factor alpha (TNFα), interleukins (IL)-1, or IL-6 and prostaglandin E_2_ (PGE_2_) could move *via* systemic circulation to distant organs (7) and contribute to cellular dysbalance far away from the side of infection. In general, this supports PD`s crosstalk and association with other chronic systemic diseases such as diabetes, obesity, cardiovascular, and musculoskeletal diseases (6, 8). Specifically, alveolar bone loss, one of the main oral features of PD, and systemic bone metabolism appear to be connected. In line with this, deterioration of lumbar vertebra, femur, and tibia bone microarchitecture, as well as aberrant levels of circulating bone metabolism biomarkers (carboxy-terminal collagen crosslinks (CTX) and parathormone (PTH)), are described in association with bacterial infection and resulting PD (8–10). Since exclusive treatment of infection has clinical shortcomings, novel preventive concepts could influence crosstalk between host response and determining cells of bone metabolism through immunomodulatory actions.

In line with this, Mediterranean-style diets (MD), high in plant-based foods, fish, and olive oil, are associated with lower prevalence and severity of inflammatory diseases such as PD (11–13). In contrast, Western-type diets (WD), high in saturated fats, processed meat, red meat, butter, and high-fat dairy products, are associated with increased inflammatory levels and susceptibility to PD (11), which is potentiated by obesity, a disease with an underlying low-grade inflammatory phenotype (11). Recent studies have explored the specific health benefits of dietary components in support of targeted non-pharmacological approaches (14, 15). Mono-unsaturated fatty acid (FA) oleic acid (OA (C18:1)) is a main constituent of olive oil-containing MDs and, in general, the most common unsaturated FA in human nutrition and serum (16). OA-serum levels correlated negatively with periodontal tissue loss (17) in patients on an optimized anti-inflammatory diet (18). In contrast, levels of saturated FAs, specifically WD-component palmitic acid (PA (C16:0)), correlated positively with PD (19).

FAs and associated bioactive metabolites show distinguished pro-inflammatory properties that could affect cellular phenotypes. Excess PA is more cytotoxic than OA (20) due to the accumulation of lipids, such as ceramide and diacylglycerols (DAG) and the induction of ER-stress, apoptosis or inflammation (21). However, OA induces triglyceride formation with no increase in pro-inflammatory markers such as TNFα (22). The lipotoxicity of PA could be counteracted by changing FA saturation (23) or converting and storing it as neutral triacylglycerols (TAGs) (20). Therefore, combined PA and OA treatment alleviated PA lipotoxicity in various cells (20, 22). OA substitution counteracted PA-induced ceramide synthesis and TNFα/IL-6-expression in hypothalamic cells, underlining the anti-inflammatory properties of OA (24). Accordingly, concomitant PA and OA treatment reduced the osteoclast-promoting impact of PA (20, 22) and converted accumulated DAG-metabolites to TAGs, thereby reducing inflammatory cellular activity in bone-forming osteoblasts (25).

In line with this, we demonstrated that obese mice on a PA-enriched diet (PA-ED) showed augmented systemic inflammatory activation and an associated disruption of bone homeostasis with increased bacterial-induced alveolar bone loss and reduced femoral microarchitecture as opposed to obese OA-fed animals (22, 26). We, furthermore, demonstrated that an OA-enriched diet (OA-ED) improved oral trabecular bone microarchitecture and enhanced circulating pro-resolving mediators Resolvin D4 (RvD4) and 4-hydroxy-docosahexaenoic acid (4-HDHA) in response to oral *P. gingivalis* inoculation in normal weight animals, whereas PA-ED did not. This underlines the clinical significance and role of systemic comorbidities as enhancers of immunometabolic pathways that determine tissue homeostasis and shape bone morphology (27). Dietary substitution of PA-ED with OA-ED increased circulating C18:1 levels and reduced the number of periodontal ligament (PdL)-surrounding alveolar bone lacunae, thereby improving alveolar bone morphology (27). Furthermore, substituted OA-ED increased bone levels of the stress-reducing phospholipid 1,2-dioleoyl-sn-glycero-3-phospho-(1’-myo-inositol) (PI(18:1/18:1)), a lipokine that is derived from mono-unsaturated FAs *via* SCD-1-activity and is involved in cell homeostasis and stress response (28) in response to oral *P. gingivalis* infection. However, whether the nutritional substitution of PA-ED with OA-ED impacts systemic bone morphology and is reflected in the bone microenvironment through the incorporation of FA-associated metabolites is not known and is of great clinical significance. In-depth information will underline dietary substitution strategies as a preventive measure and facilitate a targeted approach in support of human data, indicating the health benefits of MD as opposed to WD (12, 29–31).

We, therefore, investigated incorporated FA metabolites, their (inflammatory) impact on systemic bone homeostasis, and whether impairments are reversible by dietary substitution. Furthermore, we determined the morphological impact in response to periodontal inoculation and focused on system-wide changes in lipid modulators and cellular interactions *in vitro*.

## Methods

All procedures and evaluations were performed according to NC3Rs ARRIVE guidelines (32).

### Animals

All procedures involving animals were performed according to the German Law on the Protection of Animals/European Communities Council Directive (86/609/EEC) and approved by the Thuringia State Office for Food Safety and Consumer Protection (UKJ-17/036). Mice were maintained under appropriate barrier conditions in a 14/10hr light-dark cycle and received food and water ad libitum according to ARRIVE guidelines. Animals were housed in groups of three in IVC cages with standardized enrichment. At least two animals were housed in one cage and all cages were kept in the same room. To reduce stress, scoring, weighing, and inoculations were performed at the same time whenever possible.

### Fatty acid enriched diet

After one initial week of acclimatization, 4-week-old male C57BL/6 wildtype mice were randomly divided into groups (n=12/group) and put on either PA or OA-enriched diets (PA-ED, OA-ED) (21% calories from fat) (Ssniff, Soest, Germany) or a standard diet (ND) (11% calories from fat) for a total of 16 weeks as described before (**Table S1**) (27). Animals were assigned blinded number codes, which were used throughout the experiment. The PA-OA substitution group received PA-ED for the first 8 weeks and was placed on OA-ED afterward. Weight was monitored weekly, accompanied by scoring procedures. Thereby, we checked for weight, general condition, and general behavior. The health status of the animals was judged according to the appropriate assessment criteria defined prior to the experiments. Humane endpoints were defined based on a scoring system. The number of animals was calculated prior to the outlined experiments using power analysis (see below).

### Experimental periodontal disease

*P. gingivalis* W50 (33) (American Type Culture Collection (ATCC53978) (Manassas, VA)) was grown in defined medium as described before (33) and administered *via* oral gavage at week 10 of FA-enriched diet feeding (n=12/group). Oral inoculation was performed as described previously (27). Briefly, animals received 0.1 mg/ml enrofloxacin over their drinking water for 4 days and were infected with 109 cfu *P. gingivalis* in 50 µl of 2% methylcellulose in PBS three times a week over a period of 5 weeks then compared to the methylcellulose-treated placebo group (‘control’). Animals were inspected daily, with scorings three times a week, paralleling infections, and sacrificed one week after the final infection.

### Blood sample collection and analysis

Blood samples were taken *via* heart puncture. Samples were left clotting for approximately 2 hours at RT before being centrifuged at 5,000 x g for 10 min. Serum was collected from the supernatant and stored at -20°C. ELISA-based quantification of inflammatory proteins (Mouse IL-6 Quantikine ELISA Kit and Mouse TNF,α Quantikine ELISA Kit (both R&D Systems) or bone remodeling markers (Mouse Osteocalcin (OC) and Mouse C Terminal Telopeptide of Type I Collagen (CTX) (all supplied from MyBioSource/Biozol) in serum was performed according to manufacturer’s protocols. Serum levels were determined at an absorbance of 450 nm (Infinite M Nano plate reader, Tecan Life Science) and calculated by comparing values to a standard curve. Each sample was measured in duplicates. Protein concentrations are shown in picograms per milliliter. Each data set contained six individuals.

### Histomorphometric analysis of femoral bone

**Figure 1:**
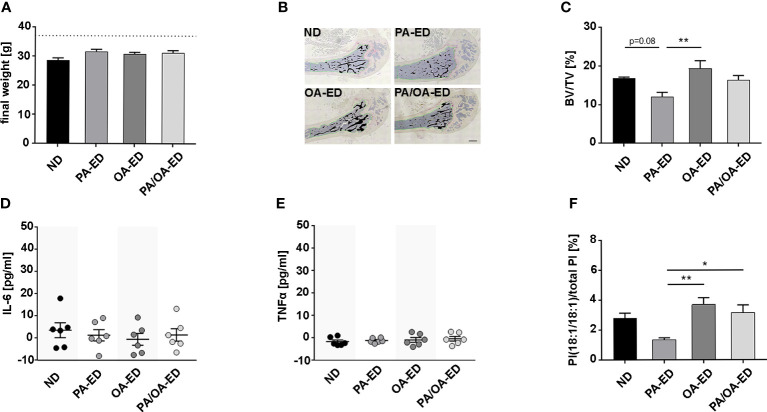
Dietary palmitic acid reduces systemic bone structure, whereas oleic-acid-enriched diet does not, which is reflected in an enhanced proportion of the stress-reducing lipokine PI(18:1/18:1) when nutritional palmitic acid is replaced with oleic acid. C57BL6 mice were fed for 16 weeks with a normal chow diet (ND), palmitic acid (PA) enriched diet (ED), oleic acid (OA)-ED, or switched from PA- to OA-ED after 8 weeks. **(A)** All animals exhibited normal weight. **(B, C)** Histomorphometric analyses of TRAP-stained femoral bones. **(B)** Representative images of TRAP-stained femoral sections (measured bone areas depicted in black, total volume measured along the green line) and **(C)** quantified as bone volume (BV) over total volume (TV) (BV/TV) (n=4). Serological levels of inflammation markers **(D)** TNFα and **(E)** IL-6 were determined by ELISA and are shown in pg/ml. **(F)** UPLC-MS/MS-based analysis of PI(18:1/18:1) in serum. The percentage values give the proportion in relation to total PI (100%). (ND normal (standard) diet, PA: PA-ED, OA: OA-ED, PA-OA group received PA-ED for the first 8 weeks and was placed on OA-ED afterward). Data are shown as mean ± S.E. Statistical analysis: ANOVA with *post hoc* test (Tukey) within treatment groups, and two-tailed Student’s t-test for pairwise comparisons. *p <.05; **p ≤ 0.01. Scale bar in B 400 µm.

Manually defleshed femurs were immersion fixed in 4% PFA overnight. Bones were decalcified in 25% EDTA for a total of 2 weeks. Paraffin slices of 4 µm were stained for tartrate-resistant acid phosphatase (TRAP) and counterstained with hematoxylin. Microscopic pictures were taken using an Axio Vert.A1 (Zeiss) using Zen software. Trabecular bone volume was measured below the growth cone using ImageJ and normalized to the tissue area (indicated by green lines in **Figure 1**) as BV/TV. TRAP^+^ cells were counted along the growth cone or a defined part of the corticalis in the diaphysis and normalized to the length. Histomorphometric data were normalized to uninfected ND-controls and shown as fold change. Each data set contained four individuals.

### PI(18:1/18:1), DAG, and TAG analysis

PIs, DAGs, and TAGs were extracted from serum or pulverized bone (34) by the successive addition of PBS pH 7.4, methanol, chloroform, and saline to a final ratio of 14:34:35:17 (35). After evaporation of the organic layer, the lipid film was dissolved in methanol and subjected to UPLC-MS/MS analysis. Lipids were separated on an Acquity UPLC BEH C8 column (1.7 μm, 2.1 × 100 mm) using an Acquity UPLC system (Waters) as described (36). The UPLC system was coupled to a QTRAP 5500 mass spectrometer (Sciex) equipped with an electrospray ionization source. DAGs and TAGs were detected based on transitions of [M + NH_4_]^+^ to [M – fatty acid anion]^+^ fragments by multiple reaction monitoring in the positive ion mode (36). PI species were analyzed in the negative ion mode by monitoring the transitions of [M-H]^-^ to both fatty acid anions (36). Relative intensities are defined as proportions of individual lipid signals (e.g., DAG (16:0/16:0)) relative to total signal intensity for a given lipid class (e.g., total DAG). Mass spectra were processed using Analyst 1.6 software (Sciex). Analysis was performed with six animals per group.

### Ceramide

Ceramide content in pulverized bone and bone marrow cells was determined using liquid chromatography coupled with triple-quadrupole mass spectrometry, as previously described (37). Pulverized bone was supplied with 1 mL H_2_O, 300 µL 6 M HCl, 1 mL methanol, 2 mL CHCl_3,_ and 10 µL of 30 µM of the internal standard N-pentadecanoyl-D-erythro-sphingosine (C15-Cer, Merck KGaA Darmstadt, Germany) in a glass centrifuge tube. The suspension was vigorously vortexed for 60 min. After centrifugation at 1900 x g for 5 min, the lower CHCl_3_ phase was transferred into a new glass centrifuge tube. After the addition of another 2 mL CHCl_3_ to the original tube, the suspension was vortexed again for 5 min and centrifuged at 1900 x g for 5 min. The CHCl_3_ phases were combined, evaporated for 50 min at 50°C in a vacuum concentrator (Christ, Osterode, Germany), and resuspended in 100 µL of a mixture of CHCl_3_ and methanol (1:4 v/v). Pelleted bone marrow cells were resuspended in 200 µL methanol after the addition of 10 µL of 30 µM of the internal standard N-pentadecanoyl-D-erythro-sphingosine (C15-Cer, Merck), vigorously vortexed for 10 min, stored at -80°C overnight, and centrifuged at 17000 x g for 5 min. The upper methanol phase was collected. Ceramides were separated using the Prominence high-performance liquid chromatography (HPLC) system consisting of the controller CBM-20A, autosampler SIL-20AC, column oven CTO-20A, pump LC-20AD, and degasser LC-20A5R (Shimadzu, Duisburg, Germany). Then, 30 µL of the samples were applied on a MultoHigh 100 RP18-3 60x2 mm column (CS Chromatographie Service GmbH, Langerwehe, Germany) maintained at 35°C under the conditions detailed in **Table S2**. Mass spectrometric determination of ceramides was performed using the QTrap triple quadrupole mass spectrometer (AB Sciex, Darmstadt, Germany) run in the multiple reaction monitoring (MRM) modus using positive atmospheric pressure chemical ionization (APCI) at 450°C. Detected single charged masses and their fragments are shown in **Table S3**.

### Isolation and cultivation of osteoblast cultures

Primary osteoblasts were isolated from long bones of the fore and hind limbs of 4-week-old C57Bl6 mice. After the removal of bone marrow, the bones were washed in PBS and cut into small pieces prior to collagenase digestion (500 U/ml Collagenase II in DMEM) for 2 h at 37°C. Bone fragments were washed three times in proliferation medium (DMEM low glucose (Thermo-Fisher Scientific), 100 U/ml penicillin, 100 µg/ml streptomycin (Thermo-Fisher Scientific), 50 µg/ml Gentamycin (Sigma-Aldrich), and 1.25 µg/ml Fungizone (Sigma-Aldrich)) and transferred to a T25-flask with 5 ml proliferation medium. The medium was changed for the first time after 5 days of incubation. Subsequent media changes were performed three times a week until cells reached confluency.

### Differentiation and infection of osteoblasts

Cells were seeded in a density of 2.8*10^4^ cells per well on 24-well plates. After 2 days in the culture, the cells reached approximately 80% confluency and the proliferation medium was exchanged for differentiation medium (proliferation medium supplemented with 100 µg/ml L-ascorbic acid, 10 nM β-glycerol phosphate, and 100 nM dexamethasone). The following day, fatty acids (FA) were added to the differentiation medium as described elsewhere (26, 38). Briefly, OA or PA was solubilized in sterile water supplemented with 50 mM NaOH at 70°C and added to prewarmed BSA (H2B) (37°C) in a 3:1 molar ratio before being transferred to the culture medium. The final FA concentration was adjusted to 0.2 mM. Medium supplemented with BSA only was used as a control. For *P. gingivalis* infection experiments, cells were cultured for a total of 6 days in differentiation medium, with one medium change after 3 days. At that time, the PA-OA group was switched from PA to OA. Prior to infection, osteoblasts were washed twice with prewarmed D-PBS (PBS without calcium, magnesium, Thermo-Fisher Scientific) and covered with 1 ml antibiotics-free culture medium (OB – DMEM low glucose with pyruvate supplemented with 10% FBS (Thermo-Fisher Scientific)). Cells were infected with a multiplicity of infection (moi) of 100 cfu in 25 µl PBS. Control cells were supplemented with 25 µl PBS. Soluble IL-6 antibody (sIL-6ab; Ultra-LEAF™ Purified anti-mouse IL-6; BioLegend) was added in a final concentration of 10 µg/ml). Application of IL-6 receptor (IL-6R; Recombinant Mouse IL-6R alpha (aa20-357) Protein, R&D Systems) was done in a concentration of 10 ng/ml (39). Cells were incubated for a total of 6 h, as described before (26). Supernatants were collected, centrifuged, and stored at -20°C until further ELISA analysis. To analyze osteoblast differentiation, cells were incubated in FA-supplemented differentiation media for a total of 14 days, with one group being changed from PA to OA treatment after 7 days. The cell culture medium was changed every 2 to 3 days.

### Osteoclast cultures

Osteoclasts were generated from the bone marrow of 4-week-old C57Bl6 mice. As described previously (40), bone marrow cells (BMC) were centrifuged out of the long bones of the front and hind limbs (10,000 x g for 20s). BMC were resuspended in proliferation medium (αMEM containing 10% FBS (Gibco) and 1% penicillin/streptomycin) and incubated overnight in 10 ml proliferation medium on a 10 cm dish (one per animal). For treatment with conditioned osteoblast media, non-adherent cells were seeded in a density of 3*10^5^ cells per well in 600 µl proliferation medium 50 ng/ml M-CSF (PeproTech) on 24-well plates. The following day, 80% of the medium was replaced by prewarmed cell culture supernatant from murine osteoblasts (FA- and PenStrep-free DMEM including 10% FBS after a 6 h secretion period) supplemented with 50 ng/ml M-CSF. sIL-6ab and/or IL-6R were added for the complete duration of supernatant incubation in a final concentration of 10 µg/ml (sIL-6ab) or 10 ng/ml (IL-6R), respectively. The cells were incubated for 2 more days prior to RNA extraction. At least three independent experiments were performed and each sample was measured in duplicate.

### Gene expression analysis

RNA was isolated with TRIzol Reagent (Thermo Fisher Scientific, Carlsbad, CA, USA)/1-bromo-3-chloropropane. Purification was performed using RNA Clean and Concentrator-5 kit (Zymo Research, Freiburg, Germany) according to the manufacturer’s instructions. The concentration and purity of RNA were measured with Nanodrop 2000 (Avantor, Radnor, PA, USA). RNA was transcribed to cDNA with SuperScript IV Reverse Transcriptase (Thermo Fisher Scientific, Carlsbad, CA, USA) according to the manufacturer’s protocol. For quantitative RT-PCR gene expression levels, Luminaris HiGreen Master Mix (Thermo Fisher Scientific) was used and monitored by qTOWER3 (Analytic JENA). Gapdh (glyceraldehyde 3-phosphate dehydrogenase) and Rps29 (40S ribosomal protein S29) served as respective reference genes. Sequences of all primers are depicted in **Table S4**. Each primer pair was tested with template dilution series for the calculation of efficiency followed by the analysis of melting curve and agarose gel electrophoresis to exclude primer dimers. Analysis of data was performed using the efficiency corrected ΔΔCT-method.

### ELISA-analysis of cell culture supernatant

Protein detection was performed using Mouse IL-6 Quantikine ELISA Kit (R&D Systems) according to the manufacturer’s protocol. Secreted IL-6 levels were calculated by comparison to a standard curve at an absorbance of 450 nm (Infinite M Nano plate reader, Tecan Life Science). At least four independent experiments were performed and each sample was measured in duplicate. Values were normalized to BSA-treated controls.

### Alizarin Red Assay

Osteoblast mineralization activity was determined using an Alizarin Red Assay (Sigma-Aldrich) according to the manufacturer’s recommendations. Briefly, at day 14, cells were fixed for 15 minutes in 10% PFA and incubated with 40 mM Alizarin Red for 20 minutes. Free Alizarin Red was washed away with water prior to cell lysis using 10% acetic acid for 30 minutes. Afterward, the suspension was heated to 85°C for 10 min and allowed to cool down for 5 min on ice. Thereafter, samples were centrifuged for 15 min at 20,000 x g and the pH of the supernatant was neutralized with 10% ammonium hydroxide. Values were determined at OD405 (Infinite M Nano plate reader, Tecan Life Science). Concentrations were determined by comparing values to a standard curve and normalized to BSA-treated controls. The experiment was repeated three times with two wells per condition.

### Power calculations/data analysis

The primary objective was to study the presence of specific lipids and bone loss in mice. Based on our previous results (26, 38), we calculated a sample size of less than six animals in each group to have a statistical power of more than 80% to detect a difference in bone loss with a significance level of 0.05 (two-tailed, STAT MAT 2.0). If not stated differently, six animals per group were analyzed.

### Statistical analyses

Comparisons within treatment groups (infected and non-infected) were analyzed using ANOVA and a *post hoc* test (Tukey) followed by a two-tailed Student’s t-test for pairwise comparisons. Analysis for differences was performed between nutrition groups (ND vs PA vs OA vs PA-OA) and infection groups vs their respective mock-infected controls. All values are depicted as mean ± SE. For the exclusion of single data points, we performed Grubb’s outlier test. We did not exclude any data points from animal experiments. Differences between groups were considered statistically significant at P < 0.05. Analyses were performed in GraphPad/InStat3 (GraphPad Software Inc., San Diego, CA, USA).

## Results

### Nutritional palmitic acid reduces systemic bone mass and alters lipotoxic stress signaling

To examine whether the nutritional intake of specific FAs mirrors previous oral findings, showing that PA compromises PDL-lining bone, mice were fed normal chow (ND), palmitic (PA)- or oleic acid (OA)-enriched diets (PA-ED, OA-ED). Additionally, a PA-to-OA dietary substitution group (PA/OA-ED) was included. All final weights were independent of dietary intake within the normal-weight range **(** **Figure 1A**). Histomorphometric analysis of TRAP-stained femur sections revealed a 38% reduced trabecular bone volume over total volume (BV/TV) in PA-ED groups compared to OA-ED groups (**Figures 1B, C**). Further, PA-ED reduced BV/TV compared to ND by approximately 30% at a p=0.08 trend. OA-ED substitution of PA-ED resulted in trabecular BV/TV not different from OA-ED and comparable to ND. Although serological IL-6- and TNFα concentrations were below inflammatory levels (41) (**Figures 1D, E**), PA-ED-associated trabecular deterioration was paralleled by significantly reduced levels of the stress-limiting lipokine PI(18:1/18:1) compared to OA-ED at the time of analysis (**Figure 1F**). Moreover, dietary substitution of PA with OA-ED significantly increased PI(18:1(18:1) compared to ND and PA-ED.

### Accumulation of saturated lipids is enhanced under a palmitic acid-rich diet and reduced by replacement with oleic acid-based diet

**Figure 2:**
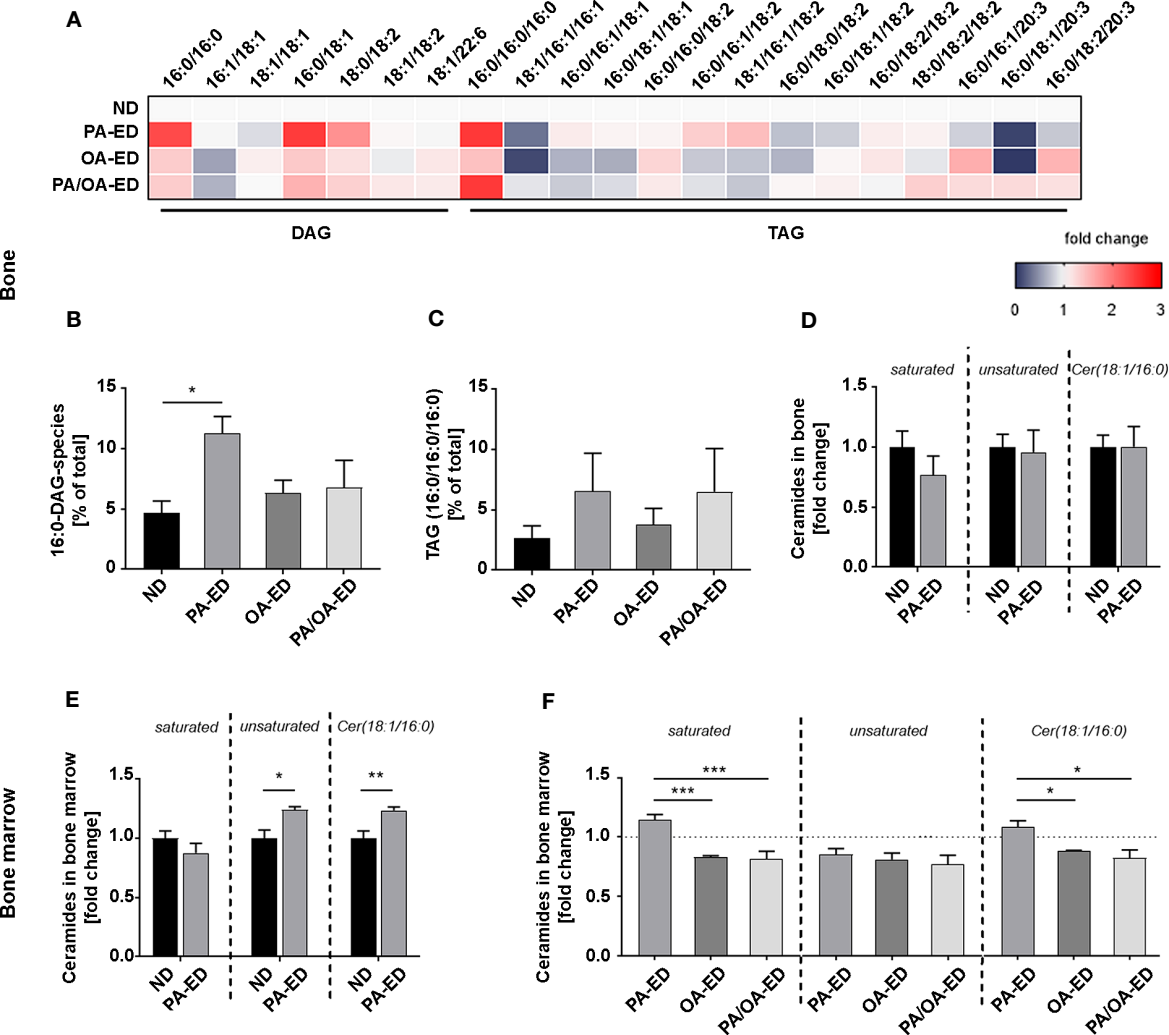
Substitution of dietary palmitic acid with oleic acid reflects in reduced bony accumulation of incorporated lipotoxic metabolites. **(A–C)** Systemic femoral bone and bone marrow of animals after 16 weeks on ND, PA-ED, OA-ED, or dietary substitution (PA/OA-ED) nutrition were analyzed by targeted lipidomics. **(A)** Heatmap showing the proportion of DAG (relative to total DAG) and TAG species (relative to total TAG) in bone, as analyzed by UPLC-MS/MS. ND is set as 100% control. **(B)** 16:0-DAG-species (DAG(16:0/16:0) and DAG(16:0/18:2)) accumulate in bone and are calculated as a percentage of total DAG (100%). **(C)** Fully saturated TAG(16:0/16:0/16:0) in bone. Values were calculated as a percentage of total TAG (100%). **(D, E)** Mass-spectrometry analyses of unsaturated (1. Panel), saturated (2. Panel), and Cer(18:1/16:0) (Cer16) ceramide-incorporations in **(D)** bone of ND and PA-ED animals and **(E)** bone marrow depicted as fold change of ND. **(F)** Unsaturated (1. Panel), saturated (2. Panel), and Cer16 ceramides in the bone marrow of FA-ED animals as fold-change of ND. ND is depicted as a dashed line. (ND: standard diet, PA: PA-ED, OA: OA-ED, PA-OA group received PA-ED for the first 8 weeks and was placed on OA-ED afterward). Data are shown as mean ± S.E. Statistical analysis: ANOVA with *post hoc* test (Tukey) within treatment groups, and two-tailed Student’s t-test for pairwise comparisons. *p <.05; **p ≤ 0.01; ***p ≤ 0.001.

To investigate whether the PA-dependent reduction and substitutional-dependent rescue in BV/TV correlate with global changes in the lipid fatty acid profile, we performed lipidomics studies on TAGs, DAGs, and ceramides from the femoral bone. PA-ED strongly elevated the relative proportion of DAG species carrying 16:0 as well as the ratio of TAG(16:0/16:0/16:0) (**Figure 2A**). The latter remained at a high level, even after nutritional OA-replacement (**Figure 2C**), whereas the accumulation of 16:0-containing DAGs decreased (**Figures 2A, B**). In consequence, substitution of PA with OA adopts a DAG/TAG profile that is more similar to OA-ED than to PA-ED, except for the fully saturated TAG(16:0/16:0/16:0).

Next, hard bone tissue and bone marrow were analyzed for PA-derived lipotoxic ceramides (**Figure 2D**). Specifically, incorporations of saturated versus unsaturated ceramides and Cer16 levels were quantified. Whereas ceramide subgroups in hard tissue were comparable, significantly more saturated ceramides and especially Cer16-ceramides (Cer18:1/16:0) were incorporated in the bone marrow of PA-ED compared to ND (**Figures 2D, E**). Overall, unsaturated ceramide levels were comparable in all FA groups. However, OA-ED by itself and the replacement of PA-ED with nutritional OA significantly lowered saturated ceramides and Cer16-content compared to PA-ED in bone marrow (**Figure 2F**).

### Oleic acid substitution abolishes palmitic acid-induced inflammatory signaling

**Figure 3:**
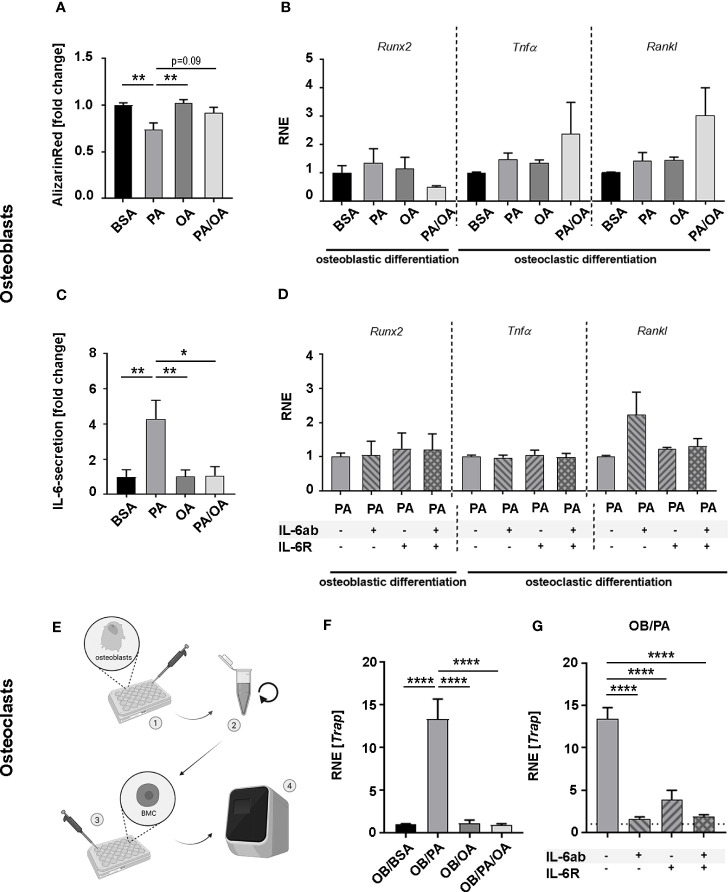
Oleic acid reverses palmitic acid-induced lipotoxic impact on osteoblasts and influences osteoblast-osteoclast interaction *in vitro*. **(A)** Osteoblast (OB) mineralization activity was analyzed in primary cultures harvested from long bones and exposed to palmitic (PA) and oleic acid (OA). Fatty acids were coupled to BSA; which also served as control. For PA-OA cultures, PA was substituted with OA after 7 days. Cells were analyzed after 14 days. Alizarin Red-staining of fatty acid (FA)-treated cells are given as fold change of BSA-cultured osteoblasts. **(B, C)** OBs were differentiated for 6 days in the presence of PA, OA, or BSA as control. PA/OA-cultures received PA after 3 days followed by 3 days of OA. Cells were incubated in FA-free media for 6 h and investigated for quantitative expression levels of osteoblastic differentiation marker *runt-related transcription factor-2* (*Runx2*), and osteoclastic differentiation factors *tumor necrosis factor-α* (*TNFα*) and *Receptor Activator of NF-κB Ligand* (*Rankl*) with relative normalized expression (RNE) displayed in relation to BSA ctrl and (C) IL-6 secretion as fold change of BSA ctrl. **(D)** OBs were differentiated under PA for a total of 6 days. Cells were incubated for 6 h in FA-free-media supplemented with interleukin-6 (IL-6) antibody (IL-6ab), IL-6-receptor (IL-6R), or a combination of both. Quantitative expression levels are displayed in relation to PA-treated cells as control and depicted as fold change. **(E)** Schematic flow chart depicting experiments (results in **F, G**) with conditioned medium from (B): • FA-treated osteoblasts were incubated in FA-free medium for 6 hours. • Cell culture supernatant was collected, centrifuged, and • applied on M-CSF-prestimulated bone marrow cells (BMCs) for 2 additional days prior to • qPCR-analysis **(F, G)**. **(F)** Trap expression levels of BMCs after incubation with osteoblastic conditioned media (OB/BSA, OB/PA, OB/OA, OB/PA/OA). Expression was calculated as fold change to OB/BSA control (relative normalized expression (RNE)) or **(G)** Trap-expression of BMCs incubated with conditioned media from PA-treated OBs supplemented with IL-6ab, IL-6R, or both. Results are depicted as fold change of OB/PA (-/-). (BSA treatment with BSA-carrier as control; OB – osteoblasts; PA – palmitic acid treatment; OA –oleic acid treatment; PA-OA-cultures were switched from PA to OA, FA – fatty acid; interleukin-6; IL-6; *Runx2*- runt-related transcription factor-2, *TNFα* - tumor necrosis factor-α; *Rankl* - Receptor Activator of NF-κB Ligand; RNE – relative normalized expression. Data are shown as mean ± S.E. Statistical analysis: ANOVA with post hoc test (Tukey) within treatment groups, and two-tailed Student's t-test for pairwise comparisons. *p < .05; **p ≤ 0.01; ****p≤ 0.0001. The graphic in **(E)** was generated with BioRender.com with an academic license.

We next investigated how specific FAs impact bone cell interaction to underline morphological findings. Osteoblasts from long bones were differentiated in the presence of FAs for 14 days. PA significantly reduced mineralization activity, whereas OA did not. Substitution of PA with OA after 7 days alleviated the impact of PA by restoring mineralization activity (**Figure 3A**).

Expression levels of osteoblastic differentiation marker (*Runx2)* or osteoclastic differentiation factors (*Rankl*, *Tnfα)* during differentiation were comparable in all groups (**Figure 3B**). However, summative IL-6 secretion during 6 hours in FA-free media after 6 days of FA-influenced differentiation was significantly enhanced in PA-cultures compared to BSA- and OA-treated cells. Change from PA- to OA-charged medium reduced IL-6 secretion significantly, resulting in levels not different from BSA- or OA-treated cells (**Figure 3C**). Neither the addition of soluble IL-6-receptor (sIL-6R) nor IL-6 scavenger antibody (IL-6ab) impacted the expression of osteoblastic and osteoclastic differentiation factors during this time frame (**Figure 3D**).

To determine the FA-specific influence on osteoblast-osteoclast interaction, we challenged pre-osteoclasts for 2 days with M-CSF-supplied FA-incubated osteoblastic culture supernatant (OB-conditioned DMEM with 10% FBS; OB/BSA, OB/PA, OB/OA or OB/PA/OA) (**Figure 3E**) and quantified the expression of the osteoclastic differentiation marker *Trap*. Whereas *Trap* expression of OB/OA-treated BMCs was comparable to controls (OB/BSA), OB/PA-treated cells exhibited a significant ~13-fold increase compared to controls. PA replacement with OA (OB/PA/OA) directly reduced *Trap*-expression levels that were now comparable to controls and OB/OA (**Figure 3F**). To verify IL-6-dependency in this context, BMCs were incubated with OB/PA-cell culture supernatant in the presence of IL-6ab, sIL-6R, or both, which significantly decreased *Trap*-expression levels in all combinations (**Figure 3G**).

### Palmitic acid augments the femoral occurrence of osteoclasts in response to oral bacterial infection and impacts cellular interaction

**Figure 4:**
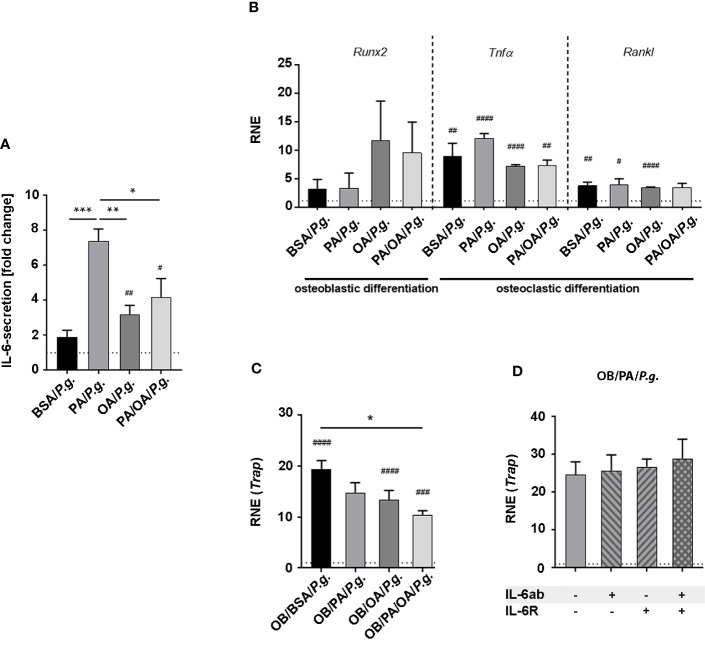
Oleic acid substitution modifies the inflammatory activation of osteoblasts and influences cellular interaction with osteoclasts in the presence of *Porphyromonas gingivalis*. **(A, B)** Osteoblasts (OBs) were incubated with fatty acid (FA)-supplemented differentiation medium (PA, OA) for 6 days. BSA-carrier alone served as control. PA/OA cultures were changed from PA to OA after 3 days. Cells were challenged with *Porphyromonas gingivalis* (*P. gingivalis*) in fatty acid (FA)-free media for 6 h and **(A)** investigated for IL-6 secretion depicted as fold change of untreated BSA ctrl (dashed line). **(B)** Furthermore, changes in expression levels in osteoblastic differentiation marker *runt-related transcription factor-2* (*Runx2*), and osteoclastic differentiation factors *tumor necrosis factor-α* (*TNFα*) and *Receptor Activator of NF-κB Ligand* (*Rankl*) were measured and displayed as relative normalized expression (RNE) in relation to BSA ctrl (depicted as dashed line). **(C)** M-CSF-pretreated bone marrow cells (BMCs) were differentiated in conditioned OB-media from **(A)** (OB/BSA, OB/PA, OB/OA, OB/PA/OA) for 2 days. *Tartrate-resistant acid phosphatase* (*Trap)*-expression was calculated as fold change of OB/BSA control (relative normalized expression (RNE), depicted as dashed line). **(D)** M-CSF-treated BMCs were challenged with conditioned media from OB from PA-treated *P. gingivalis*-infected OBs supplemented with IL-6ab, IL-6R, or both. Cells were investigated for *Trap*-expression. Results are depicted as fold change of OB/PA (-/-) (dashed line). Data are shown as mean ± S.E. Statistical analysis: ANOVA with *post hoc* test (Tukey) within treatment groups with significant differences depicted as *p <.05; **p ≤ 0.01; ***p ≤ 0.001 and two-tailed Student’s t-test for pairwise comparisons with significant changes after infection as compared to respective control displayed as #p <.05; ##p ≤ 0.01; ###p ≤ 0.001; ####p≤ 0.0001.

We next evaluated how osteoblasts respond to P. gingivalis infection under the influence of FAs. P. gingivalis incubation of FA-pretreated OBs significantly enhanced IL-6-secretion (**Figure 4A**), especially PA-stimulated secretion, compared to pretreatment conditions. After 6 days of differentiation in FA-supplied media, differentiation marker Runx2 levels were similar within FA groups and not modified by P. gingivalis infection compared to uninfected controls (**Figure 4B**, compare with **Figure 3B** for uninfected controls). Although not modified by different FA incubation, P. gingivalis infection significantly increased the expression of osteoclastic stimulators Tnfα and Rankl in almost all groups compared to the respective controls (Tnfα: BSA/ctrl vs P.g.: ^##^; PA/ctrl vs P.g.: ^####^; OA/ctrl vs P.g.: ^####^; PA/OA/ctrl vs P.g.: ^##^; Rankl: BSA/ctrl vs P.g.: ^##^; PA/ctrl vs P.g.:^#^; OA/ctrl vs P.g.: ^####^; PA/OA/ctrl vs P.g.: ns);. When M-CSF-stimulated BMCs were incubated with osteoblastic supernatant of OB/BSA- and OB/OA-treated infected and placebo-infected cells, Trap-expression increased significantly in infection groups (**Figure 4C**). Notably, the expression pattern of OB/PA/OA/P.g.-incubated cells mirrored BSA and OA-conditions (**Figure 4C**). Trap-expression of OB/PA-supernatant challenged cells was already elevated and did not increase further in response to infection. Furthermore, sIL-6R alone or in combination with IL-6ab did not change Trap-expression in PA cultures, indicating that at this time point, the process was P. gingivalis-stimulated IL-6-independent, opposite to sole PA-incubation (**Figure 4D**).

**Figure 5:**
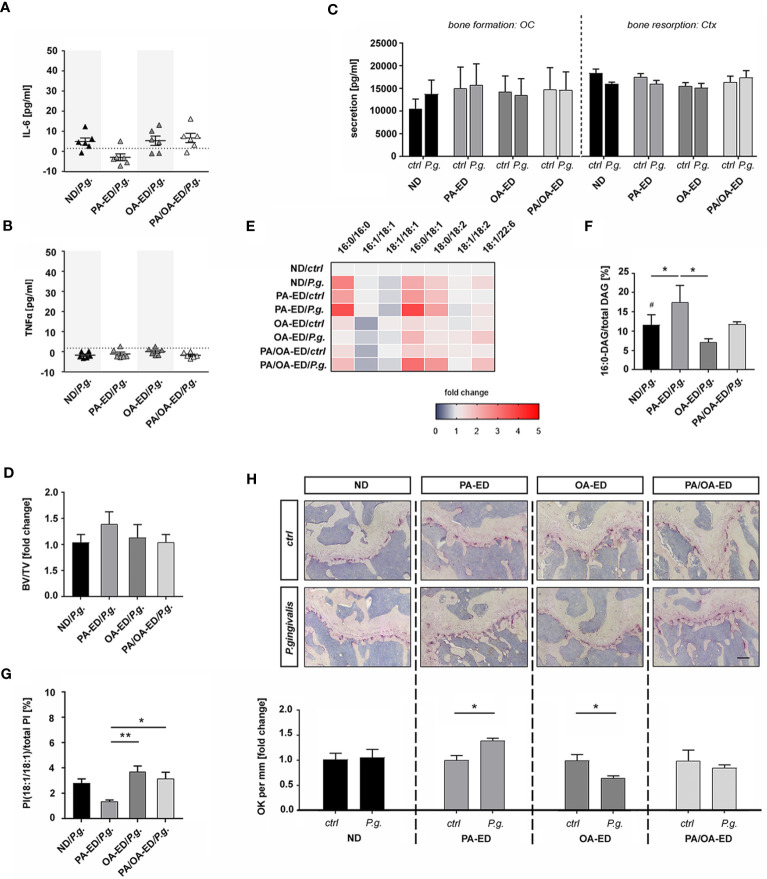
Intake of palmitic acid-enriched nutrition increases *Porphyromonas gingivalis*-induced osteoclast occurrence in the femur, whereas dietary oleic acid reduces osteoclastic occurrence. Mice were fed with fatty acid-enriched diets (palmitic acid - PA, oleic acid - OA) for 16 weeks and compared to normal chow diet (ND) animals. In one additional group PA-ED was replaced with OA-ED after 8 weeks. Animals received in total 15 oral P. gingivalis or placebo inoculations over 5 weeks. Serum levels of inflammatory cytokine **(A)** interleukin-6 (IL-6) and **(B)** tumor necrosis factor-α (TNFα) were detected using ELISA and are depicted in pg/ml with average minimum detectable doses (MDD) indicated as dashed lines (MDD IL-6: 1.6 pg/ml; TNFα: 1.88 pg/ml). **(C)** Bone remodeling markers osteocalcin (OC) and carboxy-terminal collagen crosslinks (Ctx) were determined in inoculated vs control animals using ELISA. **(D)** Histomorphometric analyses of bone volume over total volume (BV/TV) in TRAP-stained femoral sections are displayed as fold change of respective placebo controls. **(E, F)** Heatmap showing the proportion of DAG (relative to total DAG) in bone, as analyzed by UPLC-MS/MS. ND is set as 100% control. **(B)** 16:0-DAG-species (DAG(16:0/16:0) and DAG(16:0/18:2)) are calculated as a percentage of total DAG (100%). **(G)** UPLC-MS/MS-based analysis of PI(18:1/18:1) in serum. The percentage values give the proportion in relation to total PI (100%). **(H)** Differences in the presence of osteoclasts in femurs after *P. gingivalis* infection. Tissue was taken 1 week after final oral bacterial exposure. Representative images of TRAP-stained femoral sections of animals after placebo- (ctrl) or *P. gingivalis* infection. Animals had received normal diet (ND), palmitic acid (PA), or oleic acid (OA) enriched diets or a dietary substitution of PA against OA (PA-OA). Number of osteoclasts in different dietary groups with (*P. gingivalis*) and without (ctrl) infection. Values are given as fold change of the respective nutritional control and depicted as separate graphs below representative microscopic pictures. (ND: standard diet, PA: PA-ED, OA: OA-ED, PA-OA group received PA-ED for the first 8 weeks and was placed on OA-ED afterward; ctrl – placebo infection; *P. gingivalis – Porphyromonas gingivalis* infection)) Data are shown as mean ± S.E. Statistical analysis: ANOVA with post hoc test (Tukey) within treatment groups with significant differences depicted as *p < 0.05; **p ≤ 0.01 and two-tailed Student's t-test for pairwise comparisons with significant changes after infection as compared to respective control displayed as # p < 0.05. (Scale bar in PA-OA+P.g. H 200 μm).

To determine whether dietary FAs modify the systemic impact of an oral *P. gingivalis* inoculation, mice received ND, PA-ED, or OA-ED for 16 weeks. One additional PA-ED group was placed on OA-ED after 8 weeks. Animals received oral *P. gingivalis* or placebo inoculation three times a week for 5 weeks. One week after final inoculation, circulating serum IL-6 and TNFα levels were low in all groups (**Figures 5A, B**). Furthermore, the serological bone resorption marker CTX and the formation marker osteocalcin (Ocn) were not substantially altered across treatment groups (**Figure 5C**). In line with this, inoculation groups did not exhibit additional bone loss compared to the respective control groups (**Figure 5D**). However, *P. gingivalis* infection significantly elevated the proportion of 16:0-containing DAGs in the bone of ND/*P.g.* animals compared to ND/*Ctrl*. Moreover, we detected significantly increased ratios of 16:0 in DAG for PA-ED/*P.g.* compared to OA-ED/*P.g.*-treated animals (**Figures 5E, F**). Notably, serum levels of the stress-protective lipokine PI(18:1/18:1) significantly decreased upon treatment with PA-ED/*P.g.* but not by OA-ED/*P.g.* (**Figure 5G**). Switching from PA to OA substitution effectively restored the proportion of PI(18:1/18:1) within 8 weeks (**Figure 5G**). Based on these findings, we investigated the cellular presence of osteoblasts and osteoclasts in femoral bone. *P. gingivalis* inoculation did not modulate the abundance of osteocalcin-positive (Ocn^+^) cells in dietary groups (data not shown), whereas the presence of osteoclastic TRAP^+^-cells was FA-specifically modulated (**Figure 5H**). While oral periodontal inoculation in ND animals did not cause any differences between ND-control (*ctrl*) and inoculation (*P.g.*)groups (**Figure 5H**; panel 1; ND), animals on PA-ED/*P.g.* (**Figure 5H**; panel 2; PA) presented with significantly more TRAP^+^ cells compared to PA-ED/*ctrl*. In contrast, OA-ED/*P.g.* animals (**Figure 5H**; panel 3; OA-ED) showed lower amounts of osteoclasts than OA/*ctrl*. Further, dietary replacement of PA-ED to OA-ED reversed PA-lipotoxic impact, leading to TRAP-staining patterns that were comparable to controls and independent of oral inoculation (**Figure 5H**; panel 4; PA/OA).

## Discussion

Our study demonstrates that even with normal weight and in the absence of systemic inflammation, intake of the WD-component PA results in the incorporation of this fatty acid into lipids associated with cytotoxic activity (21) and an impaired systemic bone microarchitecture. Dietary substitution of PA with the major MD fatty acid OA improved trabecular femoral bone and reduced the proportion of respective metabolites. Furthermore, PA-ED modulated the systemic impact of an oral *P. gingivalis* inoculation by increasing the abundance of bone-resorbing osteoclasts in femurs. In contrast, dietary OA protected the femoral bone microenvironment by reducing osteoclast abundance, paralleled by a serological increase in the stress-reducing lipokine PI(18:1/18:1) after oral *P. gingivalis* inoculation. Of note, dietary replacement of PA-ED with OA-ED partially resolved PA-based lipotoxicity *in vivo* and in bone cell cultures in response to bacterial inoculation.

Recent studies and subsequent clinical recommendations have underlined the importance of modulating the inflammatory host response as an important clinical target in treating oral inflammatory disease (42, 43). Since nutritional intervention studies have emphasized the importance of diet composition for the inflammatory response to bacterial plaque (18, 30), recent attempts were made to classify MD- and WD-specific macro- and micronutrients and their specific impacts (44, 45). Certain fats or lipids contribute to the inflammatory status (46) and could therefore play a role locally in the periodontium and through systemic associations (11, 47). A murine study demonstrated that nutritional OA increased circulating pro-resolving lipid mediators in parallel to strengthening local alveolar trabecular bone microarchitecture in response to oral inoculation with periodontal pathogen *P. gingivalis*, contrary to WD-component PA. Although OA is one of the main components of olive oil (48), commonly used in clinical MD intervention studies (30), its anti-inflammatory capacities and positive impact on systemic health are rarely investigated in relation to oral infections. We, therefore, investigated OA in comparison to PA using a diet balanced in all other nutritional aspects and performed an analysis of associated bioactive lipid metabolites in combination with bone morphology. Findings were paralleled by the analysis of bone cell cultures, their inflammatory capacity, and their interaction to mimic the local microenvironment.

Previous investigations placed bone not only in the context of providing structural support and being a reservoir for calcium but also in the context of playing an important immunometabolic role and interacting with immune cells in a bidirectional manner (49). Nevertheless, in contrast to the large number of studies on lipid metabolism in muscle and adipose tissue, little is known about targeted FA-enriched nutrition and the occurrence of incorporated FA metabolites in relation to morphological and cellular aspects. A wide-ranging analysis of lipidomic incorporations in bone tissue revealed the presence of enhanced PA-derived lipotoxic metabolites, e.g., an increase of 16:0-DAG in hard bone and saturated ceramides, particularly Cer16, in the bone marrow of PA-ED. This was associated with reduced bone mass compared to OA-ED. Dietary substitution with OA-ED lowered lipotoxic PA-metabolites back to ND levels, accompanied by an improved bone phenotype similar to ND. Cell culture models supported these findings by demonstrating that combined PA/OA incubation alleviated PA-lipotoxicity (20, 50). Furthermore, OA treatment of PA-stimulated cells changed PA-metabolites to TAGs, counteracting PA-induced ceramide synthesis (24) and reducing inflammatory activity (25). Intracellular accumulation of PA-derived saturated ceramides and Cer16-ceramides reduced osteoblastic mineralization activity that was restored after the blockage of ceramide formation (38). Our data not only shows a PA-dependent decrease in osteoblast mineralization activity but, moreover, demonstrates that OA improved osteoblastic mineralization activity *in vitro*.

Given the contribution of inflammation to bone degradation and impaired bone formation (51), PA`s pro-inflammatory nature and aggravating impact on bone health might be based on its lipotoxic metabolites (22, 38). Cell culture data emphasized the role of inflammatory IL-6 in this context, specifically in cellular communication (52). In accordance, OA application counteracted PA-induced ceramide accumulation and associated IL-6 secretion in hypothalamic neuronal cells (24) and alleviated enhanced PA-induced IL-6 secretion levels in differentiating osteoblasts (**Figure 3**). Moreover, osteoblastic PA-stimulated IL-6 secretion directly accelerated osteoclastic differentiation from BMCs in an IL-6-dependent manner (**Figure 3**). In support, IL-6 binding to IL-6 receptors on osteoclastic precursors promotes differentiation (52). Although we could not detect differences in inflammatory serologic markers *in vivo*, we suspect, based on our *in vitro* findings and previous data, microenvironmental inflammatory changes as the driving force of the morphological bony alterations (52, 53). Additionally, distinctively reduced serum PI(18:1/18:1) levels in PA-ED indicate PA-reduced stress resistance that might influence bone loss, according to similar findings from stress-sensitive systems, e.g., infected bone under lipotoxic stress or aged hematopoietic stem cells, where PI(18:1/18:1) levels were low (28). In contrast, PI(18:1/18:1) was enhanced in OA-ED, suggesting certain stress tolerance when responding to oral bacterial infections.

Calvarial *P. gingivalis* infection caused a distant increase in osteoclast precursors in long bones (54). Notably, oral *P. gingivalis* inoculation significantly increased the femoral presence of osteoclasts in distance from the oral infection site. Our findings demonstrate PA’s ability to impact precursor cells even when overall PA intake was lower than in obese animals and without concomitant systemic inflammation. Furthermore, *P. gingivalis* infection significantly increased incorporated lipotoxic 16:0-DAGs in ND/*P.g.*, which was further enhanced in PA-ED/*P.g.* compared to OA-ED/*P.g*. Perhaps an infectious stimulus modifies FA uptake and metabolism (55). When PA-ED was replaced with OA-ED, 16:0-DAG levels were comparable to OA-ED/*P.g*., circulating PI(18:1/18:1) abundance was enhanced and the associated increase in femoral osteoclasts could not be detected anymore.

*Trap*-expression of BMCs treated with conditioned media from *P. gingivalis*-infected OBs appeared independent of IL-6, in contrast to when only PA was used as a stimulus. We speculate that a concomitant osteoblastic increase in *Rankl*- and *Tnfα*-expression might outperform and mask PA-triggered IL-6-dependent effects since RANKL and TNFα are strong osteoclastic differentiation factors by themselves (22). None of the examined FAs augmented *Tnfα* or *Rankl* expression in *P. gingivalis*-infected osteoblasts. Accordingly, *Trap* levels of OB/PA-, OB/OA, or OB/PA/OA-treated BMCs were comparable. However, the duration of FA incubation and the timing of extract collection could modify cellular interaction and secretion, as demonstrated previously (56). Cellular interactions are always a complex system, and further studies could elucidate additional mechanisms.

Local and systemic response to periodontal bacterial infection runs in stages of inflammation and resolution, which might explain the normal-range Il-6 and Tnfα serum levels in FA-ED groups. Whereas obese PA-ED-fed mice exhibited persistent systemic inflammation after *P. gingivalis* infection (26), IL-6 serum levels returned to normal 7 days after oral infection in normal-weight animals (54), underlining the importance of comorbidity control in biological systems. Of note, stress resistance as demonstrated in increased PI(18:1/18:1) levels might improve when PA-ED is replaced with OA-ED. Recent findings have described OA-ED as pro-resolving by enhancing serological RvD4 and 4-HDHA levels. Besides being pro-resolving, these lipid mediators reduced *Tnfα*-expression in gingival fibroblasts (GFb), which, downstream, could impact the microenvironment and reduce osteoclastic differentiation in GFb-co-cultures (27). Decreased osteoclast numbers in *P. gingivalis*-infected animals on OA-ED suggested that OA-intake actively impacts cells and their surrounding by advancing resolution, thereby improving bone microarchitecture in the oral cavity (27) and modifying cellular response to oral *P. gingivalis* inoculation in systemic bone.

Limitations of the study pertain to the murine periodontal inoculation model. A combination of periodontal pathogens might influence results; however, *P. gingivalis* is a PD keystone pathogen (5) and we based the current study on a previous approach (27). Further, one small initial human study supports substitution as a preventive target by demonstrating the reduction of oral inflammatory parameters after a 6-week substitution of WD with MD in normal-weight adults (30). Based on our results, future studies could investigate systemic impact in relation to nutritional components and local infection in the presence of comorbidities to address associated health problems in patient populations.

Additionally, future osteoblast-osteoclast co-culture experiments addressing FA-modulated interactions could complement the results. In the present study, we focused on FA-dependent impact on osteoblastic secretions affecting osteoclastogenesis. Since FA´s directly impact osteoclastic differentiation (22), we cultured osteoclasts in FA-free conditioned media. Tissue analysis was performed one week after the final inoculation. This might influence morphological results, the occurrence of inflammatory markers, and lipid mediators, which could be addressed in future studies. Knowledge of lipid metabolic changes in association with morphology benefits the management of other chronic inflammatory diseases with nutritional interventions in a personalized approach to therapy. Overall, oral inflammatory disease and chronic conditions share potential cross-talk genes and related pathways, as first genetic analyses have revealed (57). Similarly, our previous findings supported FA-enriched nutrition as a way to reduce generalized low-grade inflammation and combat loss of systemic bone microarchitecture in obese mice (22). Overall, our data support MD-component OA as a preventive non-pharmacological target to increase infectious stress tolerance.

## Data availability statement

The original contributions presented in the study are included in the article/**Supplementary Material**. Further inquiries can be directed to the corresponding author.

## Ethics statement

The animal study was approved by Thuringia State Office for Food Safety and Consumer Protection, UKJ-17/036. The study was conducted in accordance with the local legislation and institutional requirements.

## Author contributions

AD and US-S conceptualized and designed the study and wrote the original draft. AK contributed to the design. AD, SZ, AM, MR, MT, MHG, and US-S contributed to data acquisition. AD, SZ, AM, MR, JS, and US-S performed data analysis and, together with MHG, MW, CJ, AK, and BS, the interpretation. US-S performed the funding acquisition. All authors agree to be accountable for all aspects of the work. All authors contributed to the article and approved the submitted version.
