# Supplementary material for: Immunometabolic capacities of nutritional fatty acids in regulation of inflammatory bone cell interaction and systemic impact of periodontal infection
Source: Front Immunol. 2023 Sep 6;14:1213026. doi: 10.3389/fimmu.2023.1213026 (PMC10509849; doi:10.3389/fimmu.2023.1213026)
Supplement: Supplementary file 1 [file DataSheet_1.docx]

**Immunometabolic capacities of nutritional fatty acids in regulation of inflammatory bone cell interaction and systemic impact of periodontal infection**

**Supplemental Data**

**Figures**


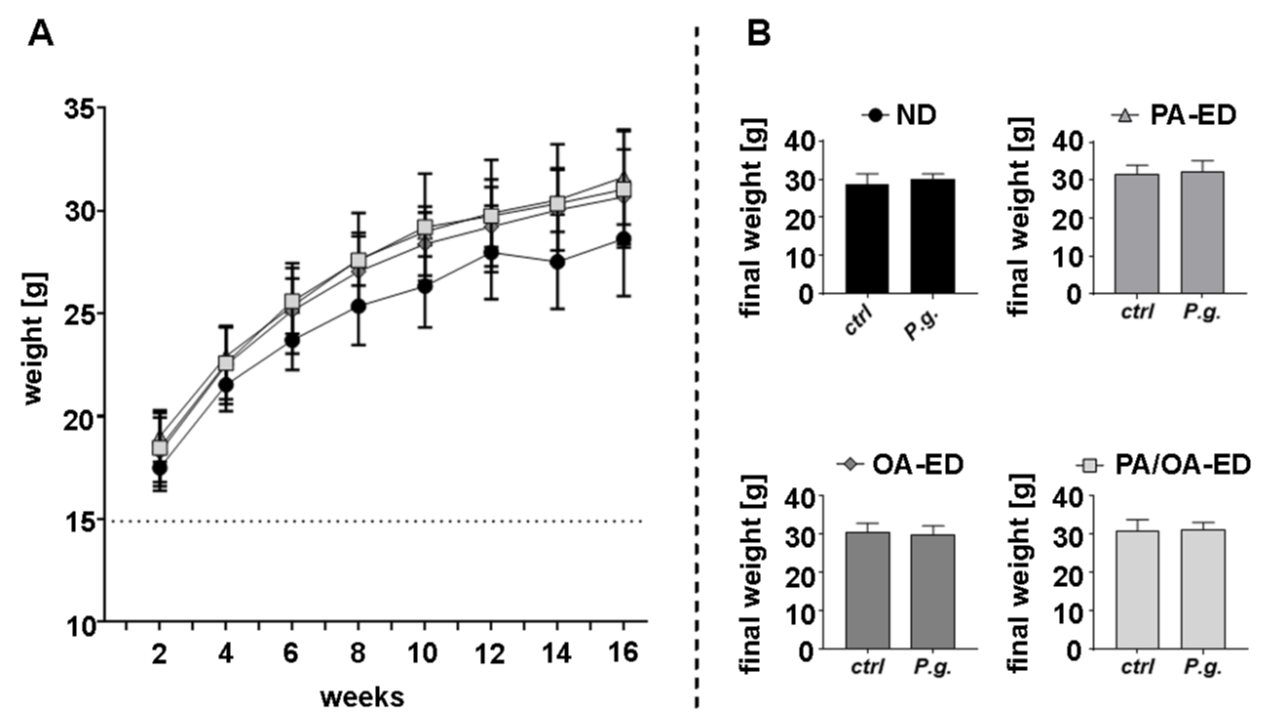


**Figure S1. Weight progression and final weights of C57Bl/6 animals. (A)** Aged 4 weeks, animals were included in the experiment. Average starting weight is given as dotted line (14.9g (Mean) (dotted line)). Black filled circle - normal diet (ND); medium grey triangle –palmitic acid enriched diet (PA-ED); dark grey diamond – oleic acid (OA)-ED; light grey square –dietary switch from PA to OA: PA/OA-ED (legend depicted as headline in respective graph in **(B)** Comparison between final weights with (P.g.) and without (ctrl) infection in different dietary groups. (ND: standard diet, PA: PA-ED, OA: OA-ED, PA-OA group received PA-ED for the first 8 weeks and was placed on OA-ED afterwards; ctrl – placebo-infection; P.gingivalis – Porphyromonas gingivalis-infection)) Data are shown as mean ± S.D.

**Tables**

**Table S1. Diet content**

|  |  | **ND** | **PA-ED** | **OA-ED** |
| --- | --- | --- | --- | --- |
|  | U | 4.1% fat | 8.6% fat | 8.6% fat |
| Casein | % | 20.00 | 20.00 | 20.00 |
| Corn-starch, pre-gelatinized | % | 36.50 | 32.00 | 32.00 |
| Maltodextrin | % | 10.49 | 10.48 | 10.49 |
| Dextrose | % | 14.00 | 14.00 | 14.00 |
| Cellulose powder | % | 7.00 | 7.00 | 9.00 |
| Inulin | % | 2.00 | 2.00 | 2.00 |
| L-Cystine | % | 0.28 | 0.28 | 0.28 |
| Vitamin premix | % | 1.00 | 1.00 | 1.00 |
| Mineral & trace element premix | % | 4.50 | 4.50 | 4.50 |
| Choline Cl (50%) | % | 0.20 | 0.20 | 0.20 |
| Dye [yellow/green/red/blue/M] | % | 0.03 | 0.04 | 0.03 |
| Palm oil | % |  | 3.80 |  |
| Palmitate ethyl ester. ≥97% | % |  | 3.80 |  |
| HO Sunflower oil | % | 3.10 |  | 7.60 |
| Soybean oil | % | 0.90 | 0.90 | 0.90 |
| **Proximate contents** | | | | |
| Crude protein | % | 17.70 | 17.70 | 17.70 |
| Crude fat | % | 4.10 | 8.60 | 8.60 |
| Crude fiber | % | 8.80 | 8.80 | 8.80 |
| Crude ash | % | 4.00 | 4.00 | 4.00 |
| Starch | % | 35.10 | 30.80 | 30.80 |
| Dextrin | % | 10.40 | 10.40 | 10.40 |
| Sugar | % | 13.90 | 13.90 | 13.90 |
| NfE (carbohydrates) | % | 60.40 | 56.00 | 56.00 |
| **Energy (Atwater)** | MJ/kg | 14.60 | 15.60 | 15.60 |
| kcal% Protein |  | 20.00 | 19.00 | 19.00 |
| kcal% Fat |  | 11.00 | 21.00 | 21.00 |
| kcal% Carbohydrates |  | 69.00 | 60.00 | 60.00 |
| **Fatty acids% in the diet** | | | | |
| C 14:0 |  | 0.01 | 0.05 | 0.01 |
| C 16:0 |  | 0.25 | 5.19 | 0.42 |
| C 18:0 |  | 0.13 | 0.22 | 0.27 |
| C 20:0 |  | 0.01 | 0.02 | 0.02 |
| C 16:1 |  | 0.01 | 0.01 | 0.01 |
| C 18:1 |  | 2.85 | 1.75 | 6.63 |
| C 20:1 |  | 0.01 | 0.01 | 0.02 |
| C 18:2 |  | 0.66 | 0.82 | 0.94 |
| C 18:3 |  | 0.06 | 0.06 | 0.06 |

Normal standard diet (ND), palmitic acid (C16:0) enriched diet (PA-ED), oleic acid (C18:1) enriched diet (OA-ED)

**Table S2.** HPLC program for separation of ceramides.

| **Time (min)** | **Flow (µl)** | **Methanol (%)** | **1 % formic acid in H_2_O** |
| --- | --- | --- | --- |
| 0.0 | 400 | 10 | 90 |
| 0.1 | 400 | 100 | 0 |
| 3.0 | 400 | 100 | 0 |
| 5.0 | 800 | 100 | 0 |
| 7.0 | 800 | 100 | 0 |
| 7.01 | 800 | 10 | 90 |
| 7.8 | 800 | 10 | 90 |
| 8.3 | 300 | 10 | 90 |
| 9.5 | 300 | 10 | 90 |
| 9.51 | 400 | 10 | 90 |
| 10.5 | 400 | 10 | 90 |

**Table S3.** Detected analytes.

| **Analyte** | **Q1** | **Q3** |
| --- | --- | --- |
| Cer (18:1/14:0) | 510,7 | 264,4 |
| Cer (18:1/15:0) | 524,7 | 264,4 |
| Cer (18:1/16:1) | 536,7 | 264,4 |
| Cer (18:1/16:0) | 538,7 | 264,4 |
| Cer (18:1/18:1) | 564,7 | 264,4 |
| Cer (18:1/18:0) | 566,7 | 264,4 |
| Cer (18:1/20:1) | 592,7 | 264,4 |
| Cer (18:1/20:0) | 594,7 | 264,4 |
| Cer (18:1/22:1) | 620,7 | 264,4 |
| Cer (18:1/22:0) | 622,7 | 264,4 |
| Cer (18:1/24:1) | 648,7 | 264,4 |
| Cer (18:1/24:0) | 650,7 | 264,4 |

Q, quadrupole; Cer, ceramide.

**Table S4.** Murine qRT-PCR-Primer used in analysis of gingival tissue and primary cell cultures

| **Gene** | **Gene**  **Symbol** | **NCBI**  **Gene ID** | **Primer Sequence (5’-3’ direction)** |
| --- | --- | --- | --- |
| Glyceraldehyde-3-phospate dehydrogenase | *Gapdh* | 14433 | Fw TGTGAACGGATTTGGCCGTA  Rev ACTGTGCCGTTGAATTTGCC |
| 40S ribosomal protein S29 | *Rps29* | 6235 | Fw GAAGTTCGGCCAGGGTTCC  Rev GAAGCCTATGTCCTTCGCGT |
| Runt-related transcription factor 2 | *Runx2* | 12393 | Fw GCCGGGAATGATGAGAAC  Rev GGACCGTCCACTGTCACTT |
| Tumor necrosis factor (ligand) superfamily, member 11 (Tnfsf11)/ Receptor Activator of NF-kB-Ligand | *Rankl* | 21943 | Fw GCAGAAGGAACTGCAACACA  Rev GATGGTGAGGTGTGCAAATG |
| Tumor necrosis factor alpha | *Tnf-α* | 51926 | Fw ACGGCATGGATCTCAAAGAC  Rev GTGGGTGAGGAGCACGTAGT |
| Tartrate-resistant-acid-phosphatase | *Trap* | 11433 | Fw CCAATGCCAAAGAGATCGCC  Rev TCTGTGCAGAGACGTTGCCAAG |
